# Factor-based reperforation timing after tympanoplasty: a graft survival analysis

**DOI:** 10.1007/s00405-025-09984-8

**Published:** 2026-02-16

**Authors:** Fatih Gül, Serkan Şerifler, Kadir Şinasi Bulut

**Affiliations:** 1 Department of Otorhinolaryngology, Lokman Hekim University Faculty of Medicine, Ankara, Turkey; 2 Department of Otorhinolaryngology, Ankara Yildirim Beyazit University Faculty of Medicine, Ankara, Turkey

**Keywords:** Tympanoplasty, Reperforation, Risk factors, Diabetes mellitus, Smoking, Time-to-event analysis

## Abstract

**Purpose:**

To investigate the impact of risk factors on the timing of reperforation (RP) after tympanoplasty and to determine the optimal follow-up intervals.

**Methods:**

This retrospective study included 403 patients who underwent tympanoplasty with at least 2 years of follow-up. The patients were divided into RP (*n* = 32) and non-RP (*n* = 371) groups. Data on age, sex, BMI, diagnosis of diabetes mellitus, smoking, atopy, perforation size and type, and middle ear status were collected based on factors previously reported to affect the graft outcomes. Group comparisons were made to identify factors associated with RP. Time-to-event analysis (Kaplan–Meier) was used to assess the effect of variables on RP timing, and linear regression was conducted to determine predictors of shorter RP time.

**Results:**

Of 403 patients, 32 experienced RP. Larger (> 50%), marginal perforations, diabetes mellitus, and smoking were significantly associated with RP. Marginal perforations (11.56 ± 5.73 months), diabetes mellitus (10.72 ± 5.09 months), and smoking (11.83 ± 4.79 months) individually led to significantly shorter RP times. The coexistence of smoking, diabetes mellitus, and marginal perforation resulted in the shortest mean RP time (6.58 ± 4.14 months). Linear regression confirmed diabetes mellitus as a significant predictor of a shorter RP time (B = -3.161, *p* = 0.049).

**Conclusion:**

Our findings show that marginal perforations, diabetes mellitus, and smoking not only increase the risk of RP, but also significantly accelerate its timing, especially when combined. This requires closer and tailored postoperative monitoring for high-risk patients to improve the long-term success of tympanoplasty.

**Level of evidence:** 4

## Introduction

Tympanoplasty is a surgical procedure aimed at repairing perforations of the tympanic membrane to restore its integrity and improve hearing function. Despite advances in surgical technology and skills, reperforation (RP) — defined as the recurrence of a perforation after initial successful closure— remains a notable complication. Various risk factors have been implicated in increasing the likelihood of RP, including age, size, and location of the perforation, status of the contralateral ear, smoking, allergic rhinitis, and the surgeon's experience [1].

The healing process of the tympanic membrane after tympanoplasty is essential in assessing the success of the procedure. The key factor to consider in this process is the successful epithelialization of the repaired perforation [2]. Numerous factors influencing the healing process have been well documented in the literature [3–5]. However, there is a lack of research examining how established risk factors influence the timing of RP following tympanoplasty, despite extensive studies regarding overall surgical success rates. Understanding the temporal aspect of RP can be essential to optimize postoperative monitoring strategies and improve patient counseling.

Follow-up periods after tympanoplasty play an important role in monitoring patient recovery and evaluating long-term success. The literature presents a range of follow-up periods, from short-term to long-term intervals, depending on the objectives of the study and the factors under investigation [6]. To our knowledge, no studies have specifically investigated the relationship between risk factors and the timing of RP. The primary objective of this study is to address the literature gap on RP timing, evaluate the impact of specific risk factors on this timing, and determine optimal follow-up intervals. In this context, we present the first analysis in the literature that focuses on the timing of RP.

## Materials and methods

### Study design and data collection

**Fig. 1:**
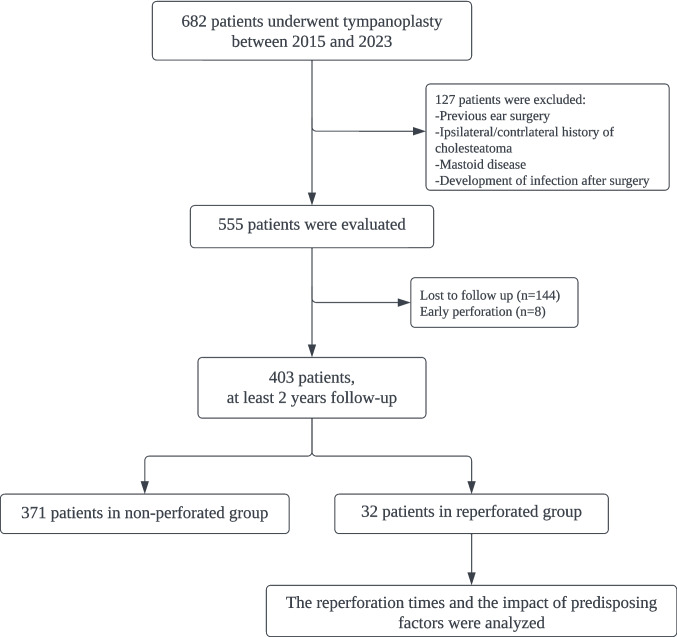
Flow diagram of the study design

A retrospective review of the chart was performed using the clinical records of 682 adult patients (aged 18 years and older) who underwent tympanoplasty between 2015 and 2023 in a university-based tertiary referral center. Formal review and approval from the local ethics committee were obtained. Inclusion criteria were patients with non-suppurative chronic otitis media, tympanic membrane perforations lasting at least six months before the surgery, and a minimum of 2 years postoperative follow-up. Exclusion criteria included patients with a history of atelectasis without perforation, otorrhea, cholesteatoma, prior ear surgeries, revision cases, and those lost to follow-up. Additionally, patients with perforations observed during the first postoperative ear examination, which were considered early perforation (EP), were also excluded from the study. The patient selection process and follow-up summary are visually represented in Fig. 1.

Medical records were reviewed to collect demographic data, including age, sex, and body mass index (BMI). Preoperative, intraoperative, and postoperative data were also collected, such as perforation size and status (marginal or central), diabetes mellitus, the last discharge from the ear before surgery, smoking, development of otitis externa after surgery, allergic rhinitis/atopy, preoperative myringosclerosis, contralateral ear status, middle ear mucosal status (mucosal edema, hypertrophy, or granulation tissue defined as an abnormal mucosa), and mastoid disease. The primary outcome was the time to RP of the tympanic membrane, which was specifically measured in months to provide a standardized and consistent assessment of the duration until the occurrence of RP.

### Surgical procedure

All operations were performed under general anesthesia using a postauricular approach under the supervision of the senior surgeon. Perichondrium with cartilage island taken from the tragus was used as graft material, placed in an underlay manner. The ossicular chain was checked for any abnormalities. After the graft was placed, the tympanomeatal flap was replaced, and the external auditory canal was packed with gel-foam soaked in antibiotic ear drops, and a mesh was placed into the external auditory canal.

### Follow-up and assessment of RPs

Patients were followed up at regular intervals postoperatively for at least 2 years. The condition of the graft had been assessed and the healing process documented for every visit in terms of perforation, graft acceptance or failure, any active disease, and signs of retraction by using a binocular operating microscope. Perforations occurring within the first two months due to factors such as graft medialization/lateralization, infection, or incomplete closure were not considered RPs; instead, they were accepted as EP. EP was defined as an early postoperative perforation occurring before the completion of epithelialization, typically due to technical or surgical issues or acute healing disturbances. RP was defined as a new perforation after initial successful graft take, with prior documented evidence of complete healing and an intact tympanic membrane, later observed during follow-up examinations.

### Explanation of methodology

In the initial analysis, factors such as diabetes mellitus, smoking status, and marginal perforation were evaluated independently, assuming that each factor was present in a single within individual patients. However, in clinical practice, these factors may coexist in the same patient. To address this, a secondary analysis was conducted to examine the RP time in cases where these factors occurred both in single and multiple. This approach allowed for a more nuanced understanding of how the interplay of multiple risk factors influences RP time. Finally, patients were categorized into three groups: single-factor, multiple-factor, and other factors.

### Statistical analysis

Statistical analyses were performed using SPSS version 30.0 (IBM Corp., Armonk, NY). Descriptive statistics were used to summarize patient demographics and clinical characteristics. Categorical variables were compared using the chi-square test, while continuous variables were analyzed using independent samples t-tests. Linear regression analysis was employed to evaluate the independent effects of diabetes mellitus, smoking, and perforation status on RP time, with the model’s overall significance determined by the F-test. A student t test was conducted to assess differences in RP times among groups with single and multiple predisposing factors, with significance set at *p* < 0.05. All results are reported with corresponding means, standard deviations, confidence intervals, and p-values.

## Results

The patient selection process and analysis of the RP and non-RP groups, as well as the impact of predisposing factors, are summarized in Fig. 1. The cohort included 32 patients in the reperforated group and 371 in the non-RP group, resulting in a reperforated rate of 7.9%. The mean age was 37.21 years, with a distribution of 54% males and 46% females. The average BMI was 23.72 kg/m^2^. In the comparison between the RP and non-RP groups, significant differences were observed in several factors. Larger perforation sizes (> 50%) were more prevalent in the RP group (*p* = 0.047). Marginal perforations were significantly associated with RP compared to central perforations (*p* = 0.012). Furthermore, diabetes mellitus (*p* = 0.022) and smoking (*p* = 0.034) were more common in the RP group. Patient demographics and clinical characteristics between the RP and non-RP groups are presented in Table 1. The mean follow-up time was 5.2 years (range, 2 to 8 years). None of the RPs healed spontaneously during the follow-up period without further intervention.

**Table 1:** Patient demographics and preoperative clinical characteristics between reperforation and non-reperforation groups

	Reperforation group, (*n* = 32)			Non-perforation group, (*n* = 371)			*p*
	*n*	mean ± SD	Adjusted residual	*n*	mean ± SD	Adjusted residual	
Age,*y*	32	38.43 ± 17.56	-	371	37.11 ± 14.81	-	0.438*
Sex							
Male	19	-	0.6	199	-	-0.6	0.58**
Female	13	-	-0.6	172	-	0.6	
Body Mass Index	32	23.11 ± 3.41	-	371	23.78 ± 3.21	-	0.764*
Mucosal status							
Normal	20	-	-1.3	272	-	1.3	0.216**
Abnormal	12	-	1.3	99	-	-1.3	
Perforation size							
< 50%	17	-	-2.1	262	-	2.1	0.047**
> 50%	15	-	2.1	109	-	-2.1	
Myringosclerosis							
Yes	13	-	1.3	111	-	-1.3	0.232**
No	19	-	-1.3	260	-	1.3	
Contrlateral Ear Status							
Non-perforated	25	-	-0.6	306	-	0.6	0.481**
Perforated	7	-	0.6	65	-	-0.6	
Perforation status							
Central	21	-	-2.8	314	-	2.8	0.012**
Marginal	11	-	2.8	57	-	-2.8	
Diabetes Mellitus							
Abscent	25	-	-2.5	340	-	2.5	0.022**
Present	7	-	2.5	31	-	-2.5	
Allergic Rhinitis							
Abscent	26	-	-1.5	333	-	1.5	0.142**
Present	6	-	1.5	38	-	-1.5	
Smoking Status							
Yes	13	-	2.1	88	-	-2.1	0.034**
No	19	-	-2.1	283	-	2.1	

^*^Student t test was used

^**^Chi-square test was used

In the RP group (*n* = 32), several factors were found to significantly influence the RP time. Patients with marginal perforations had shorter RP times compared to those with central perforations (11.56 ± 5.73 months vs. 14.68 ± 2.15 months, *p* = 0.033). Diabetes mellitus was associated with a significantly shorter RP time (10.72 ± 5.09 months) compared to those without diabetes (14.41 ± 3.30 months, *p* = 0.028). Similarly, smokers experienced shorter RP times (11.83 ± 4.79 months) compared to non-smokers (14.82 ± 2.85 months, *p* = 0.034). No significant differences were observed in RP times concerning age, sex, perforation size, mucosal status, contralateral ear status, myringosclerosis, BMI, or allergic rhinitis. These findings underscore the influence of certain clinical factors, particularly marginal perforations, diabetes mellitus, and smoking, on the time to RP, as shown in Table 2.

**Table 2:** Association of the factors and reperforation time in reperforation group

	Reperforation time, *months*, (*n* = 32)		*p*
	Mean	SD	
Age,*y*			
< 40 years	15.15	3.41	0.114
> 40 years	12.80	4.09	
Sex			
Male	13.63	3.98	0.97
Female	13.57	4.13	
Perforation status			
Central	14.68	2.15	**0.033**
Marginal	11.56	5.73	
Mucosal status			
Normal	13.03	4.65	0.29
Abnormal	14.56	2.37	
Perforation size			
< 50%	14.48	2.68	0.192
> 50%	12.62	4.99	
Myringosclerosis			
Yes	13.07	4.61	0.53
No	13.97	3.56	
Contrlateral Ear Status			
Non-perforated	13.27	4.19	0.38
Perforated	14.79	3.08	
Body Mass Index			
< 25	13.69	5.23	0.93
≥ 25	13.56	3.29	
Diabetes Mellitus			
Abscent	14.41	3.30	**0.028**
Present	10.72	5.09	
Allergic Rhinitis			
Abscent	13.43	4.20	0.61
Present	14.36	2.99	
Smoking Status			
Yes	11.83	4.79	**0.034**
No	14.82	2.85	

**Fig. 2:**
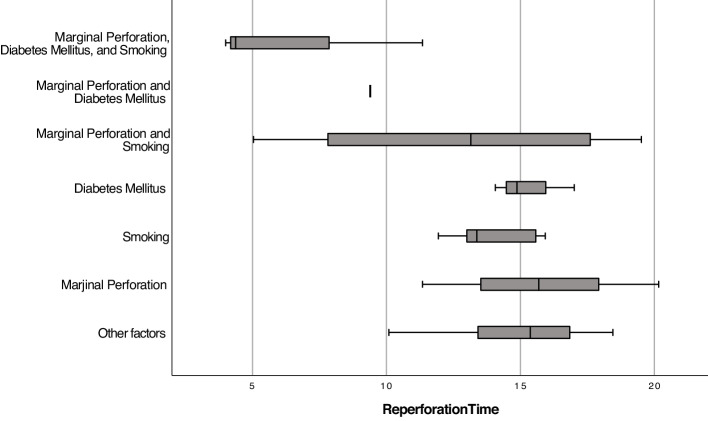
Box plot illustrating the RP times based on the presence of single or multiple risk factors

The RP times of patients with different single factors or multiple/coexisting factors present in the same patient (marginal perforation, smoking, and diabetes mellitus) were analyzed. Among single factors, the presence of marginal perforation had the longest mean RP time (15.73 ± 4.40 months), followed by diabetes mellitus (15.31 ± 1.52 months) and smoking (13.86 ± 1.55 months). For cases where multiple factors were present in the same patient, the coexistence of smoking, diabetes mellitus, and marginal perforation resulted in the shortest mean RP time (6.58 ± 4.14 months). Cases with smoking and marginal perforation had a mean RP time of 12.71 ± 6.28 months, while the presence of diabetes mellitus and marginal perforation was observed in only one case with a RP time of 9 months. A student t-test revealed a statistically significant difference in RP times between the groups (*p* = 0.018). The detailed data, including means, standard deviations, and confidence intervals, are provided in Table 3. The distribution of RP times is illustrated using a box plot in Fig. 2.

**Table 3:** Results of linear regression analysis predicting the reperforation time

	Reperforation time, *months*, (*n* = 32)									
	B*	Std. error	Beta (β)	t	*p*	95% confidence interval		Adj. R^2^	F	*p* (model)
(Constant)	15.818	0.881	-	17.962	< 0.001	14.014	17.622	0.242	4.304	**0.013**
Perforation status (Central = 0)	-1.573	1.42	-0.191	-1.107	0.278	-4.482	1.337			
Diabetes mellitus (Abscent = 0)	-3.161	1.536	-0.334	-2.059	**0.049**	-6.309	-0.016			
Smoking status (Abscent = 0)	-2.417	1.329	-0.303	-1.819	0.08	-5.139	0.306

To identify factors affecting the time to RP after tympanoplasty, a linear regression analysis was conducted. Perforation status, diabetes mellitus, and smoking status were included as independent variables to assess their predictive value on RP time. The results revealed that diabetes mellitus was a significant predictor of RP time, with patients having diabetes showing shorter times to RP (B = -3.161, *p* = 0.049). The presence of diabetes mellitus significantly reduces this baseline RP time by 3.161 months, highlighting its substantial impact on the overall model. Although smoking status showed a reduction in RP time, it did not reach statistical significance (B = -2.417, *p* = 0.08). Additionally, the perforation location did not significantly affect RP time (B = -1.573, *p* = 0.278). The overall model explained 24.2% of the variance in RP time and was statistically significant (*p* = 0.013). These findings are summarized in Table 4.

**Table 4:** Reperforation time data for single and multiple factors (Marginal perforation, smoking, diabetes mellitus, and coexistence cases)

		Reperforation time, *months*, (*n* = 32)				
		*n*	Mean ± SD	95% Confidence interval		p*
				Lower	Upper	
Single factor		12	14.69 ± 2.41	13.16	16.23	**0.018**
	Presence of Diabetes Mellitus	3	15.31 ± 1.52	11.54	19.09	
	Presence of Smoking	6	13.86 ± 1.55	12.23	15.50	
	Presence of Marginal Perforation	3	15.73 ± 4.40	4.79	26.67	
Multiple factors		8	9.99 ± 5.57	5.33	14.66	
	Presence of Diabetes Mellitus and Marginal Perforation	1	9	-	-	
	Presence of Smoking and Marginal Perforation	4	12.71 ± 6.28	2.70	22.71	
	Presence of Smoking, Diabetes Mellitus, and Marginal Perforation	3	6.58 ± 4.14	-3.71	16.86	
Other non-significant factors (single or multiple)		12	14.92 ± 2.52	13.32	16.53	**-**

^*^Student t test was performed between single factor group and multiple factors group

## Discussion

Tympanoplasty remains a cornerstone procedure for restoring tympanic membrane integrity and hearing function; however, RP, defined as the recurrence of a perforation after initial successful closure, represents a significant complication. Despite advancements in surgical techniques and a wealth of research identifying various risk factors for RP, there has been a lack of studies specifically investigating how these established risk factors influence the timing of RP. Our study sought to address this critical gap by conducting the first analysis in the literature focusing on the temporal aspect of RP in relation to specific risk factors, aiming to optimize postoperative monitoring strategies and patient counseling.

Complete healing and epithelialization of the reconstructed tympanic membrane typically occur within the first few months following successful tympanoplasty, especially in patients without comorbidities or conditions that impair healing. In line with this, we observed that 144 out of 555 eligible patients did not return for follow-up and were excluded from the study, reflecting a common clinical reality: patients with uneventful recoveries often do not attend long-term postoperative visits. However, we believe that long-term follow-up should not be entirely dismissed, particularly in selected patient populations. The mechanical stability of cartilage may mask underlying vulnerabilities in the early period, while long-term integration may still fail. Therefore, we advocate for risk-based follow-up, with extended monitoring (up to 12–18 months) recommended for patients with elevated long-term complication risk.

### Factors influencing RP

Concomitant diseases and risk factors such as perioperative infections, diabetes mellitus, smoking may not impact EP or epidermization because full-thickness cartilage grafts offer mechanical stability and are less dependent on immediate revascularization for survival. Their rigid structure provides durable support during the early postoperative phase, protecting the graft from biological stress and suboptimal healing conditions. This rigidity effectively “buys time” for epithelialization and integration, explaining why these risk factors primarily influence RP in the longer-term follow-up rather than causing early failure. Similar to how cartilage grafts protect against early failure in patients with systemic risk factors, their mechanical stability also helps buffer the effects of conditions like eustachian tube dysfunction and middle ear ventilation issues.

Our findings corroborate previously reported factors associated with the occurrence of RP [1, 7]. Consistent with existing literature, our retrospective analysis of 403 patients revealed that larger perforation sizes (> 50%), marginal perforations, diabetes mellitus, and smoking were significantly more prevalent in the RP group (*p* < 0.05 for all). These observations align with the understanding that compromised healing environments, vascular supply, and tissue quality, often associated with these conditions, can impede successful graft integration and epithelization.

The novel contribution of this study lies in its focus on the timing of RP. Our time-to-event analysis highlighted that marginal perforations, diabetes mellitus, and smoking were individually associated with significantly shorter RP times (*p* < 0.05 for all). Specifically, patients with marginal perforations reperforated earlier (11.56 ± 5.73 months) compared to central perforations (14.68 ± 2.15 months). Similarly, diabetic patients experienced shorter RP times (10.72 ± 5.09 months) than non-diabetics (14.41 ± 3.30 months), and smokers reperforated more quickly (11.83 ± 4.79 months) than non-smokers (14.82 ± 2.85 months). Findings underscore the profound influence of these specific clinical factors on the rapidity of graft breakdown. Interestingly, other commonly cited factors such as age, sex, and BMI did not show a significant impact on RP timing in our cohort.

### Literature review on the timing of RP

In the literature, there are very few studies that differentiate between RP and EP. In a 20-year follow-up study including 359 patients, tympanoplasty outcomes were shown to decline over time, with an initial success rate of 94% decreasing to 92% due to delayed RPs. The follow-up period ranged from 6 months to 20 years, with a median of 47 months, highlighting the importance of long-term monitoring and the distinction between early and late graft failure [8]. A major gap in the existing literature is the lack of studies investigating the relationship between the timing of RP and predisposing factors. This gap highlights the originality and contribution of our study.

Noh et al. conducted a study that could be similar to timing by using vascularization time, but only the onset of vascularization time was considered here [9]. In their study, vascularization occured earlier in small perforations than in large perforations of > 90%. In our study, earlier RP was observed in cases with larger perforations. This finding may be attributed to delayed vascularization, which could impair graft nutrition and lead to necrosis.

In a prospective database study including 837 cases, the graft take-rate was reported as 93.0% at 2 to 6 months and 86.6% at more than 12 months [10]. In a prospective audit study of 1070 patients, failure rates increased significantly over time, with higher rates at 6–12 months compared to 3–6 months, and at 3–6 months compared to 1–2 months [11]. In a study of 182 cases, 30 failures were reported, with 63% of RPs occurring within the first month, 23% within three months, and the remaining 14% within 20 months, while no RPs were observed beyond 20 months during a 68-month mean follow-up [12]. A recent meta-analysis showed that although longer follow-up periods were associated with increased failure rates, no significant correlation was found between follow-up duration and surgical outcome. Notably, studies with follow-up periods exceeding 12 months demonstrated higher failure rates (≤ 6 months: 87.15%, ≤ 12 months: 85.61%, > 12 months: 82.77%) [13]. In our study, however, the timing of RP appeared to be influenced by multiple patient-related factors such as smoking, diabetes, and marginal perforation.

### RP time in single and multiple predisposing factors

A particularly crucial finding from our analysis was the synergistic effect of multiple risk factors. The analysis revealed that the coexistence of multiple factors, such as smoking, diabetes mellitus, and marginal perforation, significantly shortened the RP time, underscoring the cumulative impact of these risk factors. The presented data in Table 4 offer a preliminary understanding of the relationships between these single/multiple factors and RP time, although there are both strengths and limitations to consider. Interestingly, evaluated as single factors, diabetes mellitus alone was associated with a mean RP time of 15.31 months (*n* = 3), marginal perforation with 15.73 months (*n* = 3), and smoking alone with a slightly shorter time of 13.86 months (*n* = 6). When the coexistence of these factors, the data suggest that multiple risk factors may exacerbate the condition, leading to shorter RP times. The coexistence of smoking, diabetes mellitus, and marginal perforation results in a markedly reduced mean RP time of 6.58 months (*n* = 3), indicating a synergistic effect of these factors. This suggests that the interplay between these factors exacerbates the risk of early RP, likely due to their combined detrimental effects on healing and tissue integrity.

Despite focusing primarily on the three most prominent risk factors—diabetes mellitus, smoking, and marginal perforation—it is important to acknowledge that other unmeasured or non-significant variables may also influence reperforation timing. While some patients classified under “single” or “multiple” risk factor groups might have had additional conditions (e.g., mucosal status, contralateral ear findings, allergic rhinitis), these were not included in the core subgroup analysis due to their lack of statistical significance. This is further supported by our linear regression model, which explained only 24.2% of the variance in RP time (*p* = 0.013), indicating that other biological or environmental contributors remain unaccounted for. Notably, recent experimental data by Zhang et al. demonstrated that impaired neovascularization could lead to incomplete tympanic membrane healing despite adequate epithelial migration, due to the failure of fibrovascular support formation [14]. These findings suggest that RP is a multifactorial outcome, and future research should consider molecular and tissue-level markers to more comprehensively assess healing dynamics and RP risk.

### Predictive factors for RP timing

Our linear regression analysis further reinforced the predictive power of these factors on RP timing. Diabetes mellitus emerged as a significant independent predictor of shorter RP time (B = -3.161, *p* = 0.049), indicating that the presence of diabetes significantly reduces the baseline time to RP by approximately 3.16 months. Although smoking also showed a trend towards reducing RP time, it did not reach statistical significance in this model (B = -2.417, *p* = 0.08). The overall model was statistically significant (*p* = 0.013) and explained 24.2% of the variance in RP time, suggesting that other unexamined factors or their complex interactions also contribute to the timing of RP.

### Rationale for the first two-month cutoff in defining RP

The tympanic membrane's healing process relies on the perforation size and the epithelial cell migration rate. A recent review found that the mean rate of epithelial migration in the healthy tympanic membrane is 94.51 μm per day [2]. Due to radial migration, two opposing edges close simultaneously, effectively doubling the migration speed to 189.02 microns per day. Considering that the perforation is enlarged during the desepithelialization of the perforation borders, the perforation area was considered to be up to 8–9 mm, which is the largest possible size for the membrane. Thus, epithelialization of a perforation measuring 8–9 mm could take approximately 42 to 48 days. A two-month follow-up period was used in our study to ensure adequate healing and to define the upper limit for identifying EP. This approach is also supported by previous findings indicating that approximately 90% of grafts appear fully healed by the 6-week postoperative visit [15]. In a previous study, a three-month cutoff was used to differentiate between graft failure/residual perforation (before three months) and late or recurrent perforation (after three months); however, it is noteworthy that this timeframe was not supported by a clearly defined physiological or evidence-based rationale [16]. Notably, in our cohort, the earliest observed RP occurred at 4 months postoperatively.

Numerous studies confirm that neovascularization is a fundamental prerequisite for graft integration and successful tympanic membrane regeneration. Applebaum and Deutsch, using fluorescein angiography, demonstrated that vascular ingrowth into temporalis fascia grafts typically begins within the first 1–2 weeks postoperatively and is generally completed by 3–4 weeks, progressing from the annulus and mallear regions toward the center [17]. These findings have been supported by Noh and Lee, who reported a mean vascularization time of approximately 14 days, with significantly delayed revascularization observed in larger or subtotal perforations [9]. The size of the perforation directly affects the timeline of both vascularization and epithelialization. As Wu and Lou observed in an endoscopic clinical study, smaller perforations show early peripheral vascular bridging, often completing integration by the third postoperative week, whereas larger defects may take up to 4 weeks or more for complete revascularization, with epithelial migration similarly delayed [18].

## Limitations

A limitation of this study is that the follow-up visits were not standardized, resulting in varying time intervals between appointments. When RP was detected, the actual development time of the RP may have been earlier than the time of observation. To address this limitation and standardize the time measurement, the RP time was recorded in months rather than weeks. Another limitation of this study is the lack of a clear distinction in the literature between EP and RP, which creates ambiguity in determining the appropriate time to assess and define RP. To address this uncertainty, we excluded cases of perforation observed during the first two months after the surgery. Additionally, a significant limitation of this analysis is the small sample size, particularly in groups with multiple risk factors, which is inherent to the nature of the study given the low rates of RP. For instance, the group with both diabetes mellitus and marginal perforation consists of only one case (*n* = 1), which limits the generalization and statistical power of the findings. The small sample size also precludes the use of post hoc tests, further restricting the ability to draw definitive conclusions. Moreover, although a 24-month follow-up period is generally considered sufficient in the literature to assess long-term tympanoplasty outcomes, evidence from studies with extended follow-up durations suggests that a small proportion of delayed RPs may still occur beyond this period.

## Conclusion

Our study provides crucial evidence that marginal perforations, diabetes mellitus, and smoking not only increase the likelihood of RP but also significantly shorten the time until it occurs, particularly when these factors are present concurrently. These findings advocate for differentiated and intensified postoperative monitoring schedules for high-risk patients, ultimately improving the long-term success of tympanoplasty.

## Data Availability

The datasets generated and/or analyzed during the current study are not publicly available due to patient confidentiality and institutional regulations but are available from the corresponding author on reasonable request.
